# Rediscovery of *Orientotlosiishibai* Sakai, 1980 (Crustacea, Decapoda, Brachyura, Leucosiidae) in Taiwan

**DOI:** 10.3897/zookeys.1053.67326

**Published:** 2021-08-02

**Authors:** Peter K. L. Ng, Tin-Yam Chan

**Affiliations:** 1 Lee Kong Chian Natural History Museum, National University of Singapore, 2 Conservatory Drive, Singapore 117377, Singapore National University of Singapore Singapore Singapore; 2 Institute of Marine Biology and Center of Excellence for the Oceans, National Taiwan Ocean University, Keelung 202301, Taiwan National Taiwan Ocean University Keelung Taiwan

**Keywords:** East Asia, Leucosioidea, new record, redescription, rubble crab, taxonomy

## Abstract

The leucosiid crab *Orientotlosiishibai* Sakai, 1980 was described from one female collected off western Japan and had never been reported since. The species is now recorded from southwestern Taiwan for the first time, and is redescribed and figured at length. Although Sakai argued that *Orientotlos* Sakai, 1980, is closely related to *Oreophorus* Rüppell, 1830 and *Atlantotlos* Doflein, 1904, the genus is actually morphologically most similar to *Merocryptus* A. Milne-Edwards, 1873. The two genera, however, still differ markedly in a number of key carapace and cheliped characters.

## Introduction

Sakai (1980) described an unusual new genus and new species of leucosiid crab, *Orientotlosiishibai* Sakai, 1980, from a single female obtained from trawl bycatch from off Kumano-nada, Mie Prefecture, on the Pacific side of central Japan. Sakai (1980) commented that it was closely related to *Oreophorus* Rüppell, 1830, and *Atlantotlos* Doflein, 1904, but differed in carapace and cheliped features. The genus had never been reported since its description. We here report a specimen of *Orientotlosiishibai* recently collected in southern Taiwan. The species is redescribed and figured, and its taxonomic position is discussed.

## Material and methods

The terminology used follows Tan and Ng (1996), with amendments by Davie et al. (2015). Measurements provided are of the maximum carapace width and length, respectively. The specimen is deposited in the National Taiwan Ocean University (**NTOU**), Keelung, Taiwan.

## Taxonomy

### Family Leucosiidae Samouelle, 1819

#### Genus Orientotlos

Taxon classificationAnimaliaDecapodaLeucosiidae

Sakai, 1980

65390596-6814-5134-B5E4-73D5D99F14CA

##### Type species.

*Orientotlosiishibai* Sakai, 1980, by original designation.

##### Diagnosis.

Carapace subhexagonal in outline; dorsal surfaces between plates and bosses with numerous, well-spaced boletiform and rounded tubercles; subhepatic region forming a distinct obtuse angle visible in dorsal view; hepatic plate distinct, separated from first anterolateral tooth by wide cleft; anterolateral margin with 3 large lobiform teeth; posterolateral margin concave, with median triangular tooth; posterior carapace margin with 2 large lozenge-shaped bosses; postfrontal median keel prominent, high, extending posteriorly to cardiac region as raised row of rounded tubercles; postorbital region without deep depression; large boletiform plates on protogastric, epibranchial and metabranchial regions; cardiac region with raised, vaguely T-shaped ridge formed of fused granules; intestinal region inflated, with a large subtriangular boletiform plate; antennule with basal segment occupying lower two-thirds of fossa; basal antennal article large, subquadrate, fused with epistome, forming most of suborbital margin; third maxilliped with merus, ischium and exopod paved with numerous flattened rounded tubercles, basal parts with boletiform tubercles, exopod stout, broad, reaching to about half length of merus; palm of cheliped short, stout, without ridges, lobes or teeth, fingers shorter than palm; ambulatory legs short, merus, carpus and propodus covered with slender and boletiform tubercles along upper and lower margins, dactylo-propodal lock present; anterior thoracic sternites (1–4) strongly compressed, surface of sternite 3 with numerous boletiform tubercles; female thoracic sternite 4 forming keel around distal part of sternopleonal cavity; vulvae relatively small, round, positioned distinctly apart; female pleon ovate, shield-like, covered with rounded tubercles, somites 1 and 2 free, somites 3–6 fused, telson narrowly triangular with distal part linguiform.

##### Remarks.

The concept of *Oreophorus* Rüppell, 1830 has changed substantially since 1980, with several revisions clarifying the identities of allied genera (*Tlos* Adams & White, 1849, and *Oreotlos* Ihle, 1918) and the establishment of several new ones: *Dolos* Tan & Richer de Forges, 1993, *Alox* Tan & Ng, 1996, and *Cateios* Tan & Ng, 1996. *Orientotlos* can nevertheless be easily distinguished from these genera by the anterolateral margin of carapace not expanded posteriorly and sometimes reaching level of the posterior carapace margin, the anterolateral margin is distinctly lobiform or dentiform, dorsal carapace surface without regions distinctly raised to form bosses, with depressed areas never eroded and no obvious postocular depression or groove, cardiac region not large or strongly inflated, and the palm of cheliped is short ovate, with fingers short and relatively slender and cutting edges lined with low teeth (cf. Tan and Richer de Forges 1993; Tan and Ng 1996).

The carapace of *Orientotlos* only superficially resembles that of *Atlantotlos* (type and only species *Atlantotlosrhombifer* Doflein, 1904), described from off the Congo in West Africa, in general shape (Doflein 1904: 51). The latter genus differs markedly from *Orientotlos* in possessing a smooth carapace, without inflated bosses, boletiform, or rounded tubercles, an entire posterior carapace margin, and less prominently armed pereopods (cf. Doflein 1904: pl. 15, figs 7, 8).

**Figure 1.**
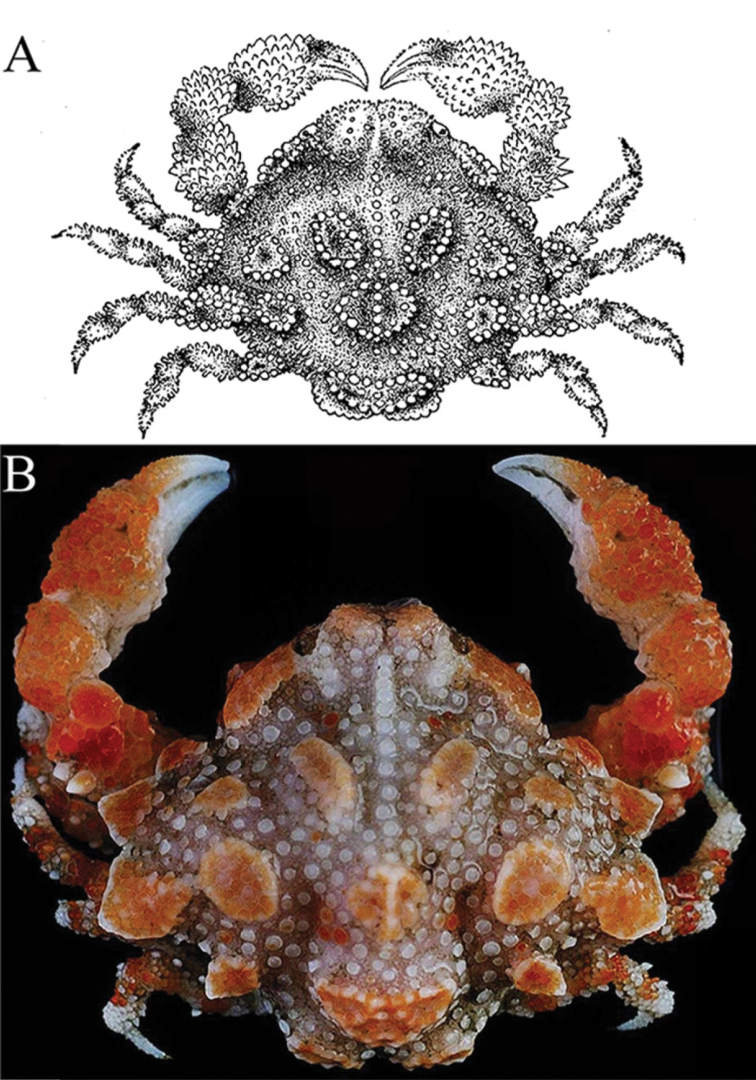
*Orientotlosiishibai* Sakai, 1980 **A** holotype female (7.0 by 5.5 mm) (repository unknown), Japan [after Sakai 1980: text-fig. 1] **B** color in life, female (7.8 × 6.5 mm) (NTOU B00125), Taiwan.

**Figure 2.**
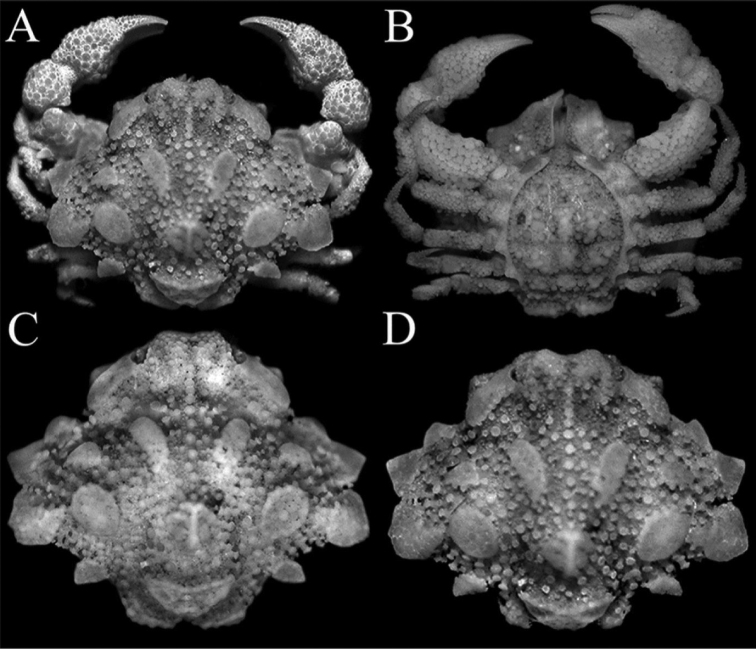
*Orientotlosiishibai* Sakai, 1980, female (7.8 × 6.5 mm) (NTOU B00125), Taiwan **A** overall dorsal view **B** overall ventral view **C, D** dorsal view of carapace from different angles.

**Figure 3.**
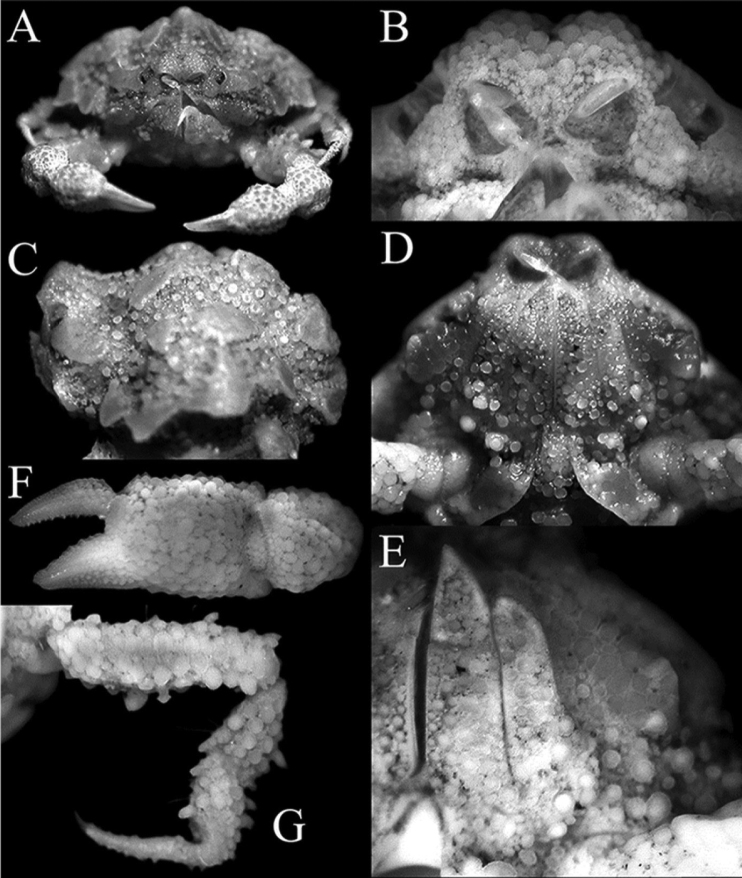
*Orientotlosiishibai* Sakai, 1980, female (7.8 × 6.5 mm) (NTOU B00125), Taiwan **A** frontal view of cephalothorax **B** frontal view showing antennules, antennae and orbits **C** lateral view of cephalothorax **D** pterygostomial region, buccal cavity, third maxillipeds and anterior part of sternopleonal cavity **E** left third maxilliped **F** outer view of left chela **G** right fourth ambulatory leg.

**Figure 4.**
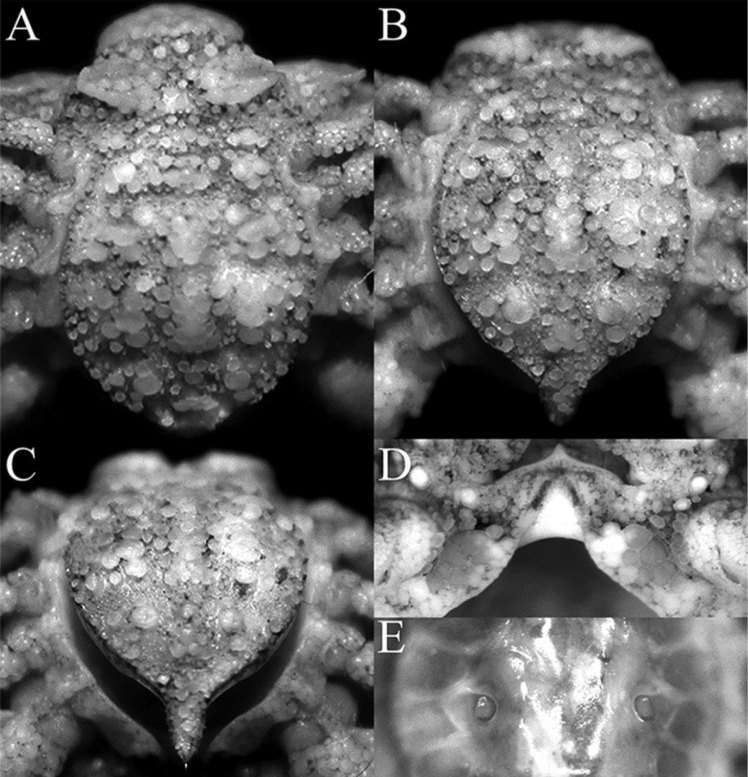
*Orientotlosiishibai* Sakai, 1980, female (7.8 × 6.5 mm) (NTOU B00125), Taiwan **A** intestinal region, posterior carapace lobes and pleonal somites 2–6 **B** posterior carapace lobes, pleonal somites 2–6, and telson **C** pleonal somites 4–6 and telson **D** anterior thoracic sternites and sternopleonal cavity **E** sternopleonal cavity and vulvae.

*Orientotlos* is actually most similar to *Merocryptus* A. Milne-Edwards, 1873 in having the anterolateral margin of the carapace with three large lobiform teeth (Figs 1, 2A, C, D) (versus margin with low lobes or spines but never clearly dentiform); a prominent lobe on posterolateral margin of carapace (Figs 1, 2A, C, D) (versus lobe absent or undiscernible in *Merocryptus*, although margin may be uneven or spiniform); median keel on gastric, cardiac and intestinal regions high and strongly inflated (Figs 1, 2A, C, D) (versus much lower in *Merocryptus*); intestinal region formed by a pair of distinct, fused subtriangular bosses (Figs 1, 2A, C, D) (versus more coniform or evenly rounded in *Merocryptus*); posterior carapace margin with two broad truncate, lozenge-shaped bosses (Figs 1, 2C, D) (versus entire or with dentiform projections in *Merocryptus*); female cheliped palm and fingers proportionately shorter and stouter (Figs 1, 2A, B, 3F) (versus more elongate and slender in *Merocryptus*); and vulvae widely spaced (Fig. 4E) (versus proportionately larger and closer to the midline in *Merocryptus*) (cf. A. Milne-Edwards 1873; Yokoya 1933; Serène 1955; Sakai 1976; Zarenkov 1994; Chen and Sun 2002; Galil 2019; Galil and Ng in press).

#### Orientotlos iishibai

Taxon classificationAnimaliaDecapodaLeucosiidae

Sakai, 1980

BFBD7B01-F0A1-5F6B-BCBB-EE3FB81EC14A

Figs 1 , 2 , 3 , 4

Orientotlos iishibai Sakai, 1980: 74, text-fig. 1. – Ng et al. 2008: 92 (list).

##### Material examined.

Taiwan • 1 ♀ ovigerous (7.8 × 6.5 mm); station CP4210, off southwestern Taiwan coast; 22°18.94'N, 120°20.57'E; depth 116–159 m; 14 Nov. 2020; T.-Y. Chan leg.; hard bottom substrate; NTOU B00125.

##### Diagnosis.

As for genus.

##### Description.

**Female.** Carapace subhexagonal in outline, 1.2× as wide as long; upper surface (between plates and bosses) paved with numerous, well-spaced boletiform and rounded tubercles of varying sizes (Figs 1, 2A–D). Front produced, upturned, margin weakly bilobed with shallow median concavity; frontal margin gently confluent with concave supraorbital margin; hepatic region plate-like, formed by coalesced granules; subhepatic region swollen, forming a distinct obtuse angle visible in dorsal view; hepatic plate separated from first anterolateral tooth by a wide, deep cleft; anterolateral margin with 3 large lobiform teeth increasing in size posteriorly, third tooth directed obliquely, surface paved with flattened granules, margins lined with low granules; posterolateral margin concave, with median triangular tooth, margin with rounded granules; posterior carapace margin with 2 large lozenge-shaped bosses, directed posteriorly, margin flattened, surface and margins lined with rounded and flattened granules (Figs 1, 2A–D). Suborbital region substantially compressed; subhepatic region swollen, surface with large, flattened tubercles, margins of subhepatic and pterygostomial regions covered with numerous boletiform tubercles (Fig. 3D, E); subhepatic and hepatic regions separated by distinct groove lined with granules, inner edge leading to just before tip of efferent branchial channel, outer edge joining cleft between hepatic lobe and first anterolateral tooth (Fig. 3A, B). Postfrontal median keel on gastric region prominent, high, extending posteriorly to cardiac region as raised row of rounded tubercles; postorbital region gently concave, without deep depression; median part of carapace dome-shaped, protogastric region with ovate boletiform plate, obliquely positioned, margin granulated; cardiac region swollen, with raised, vaguely T-shaped ridge formed of fused granules surrounded by rounded granules; epibranchial region with a small, subovate, transversely positioned boletiform plate, margin lined with small granules; mesobranchial region with large ovate boletiform plate, margin lined with granules; intestinal region strongly inflated, with a large subtriangular boletiform plate, directed posteriorly, margin with distinct granules (Figs 1, 2A, C, D, 3A, C). Orbital margin not clearly marked, gradually merging with granules from frontal margin and hepatic region; cornea visible in dorsal view, peduncle short with small sharp granules (Fig. 2C, D). Antennule folded into an oblique fossa; basal segment finely granulate, occupying lower two-thirds of fossa (Fig. 3B). Basal antennal article large, subquadrate, fused with epistome, forming most of suborbital margin, covered with rounded tubercles; antennae small, slender, inserted in orbital hiatus (Fig. 3B). Anterior margin of efferent branchial channel produced, reaching to just before proepistome, slightly notched (Fig. 3B).

Third maxilliped with merus, ischium and exopod paved with numerous flattened, rounded tubercles of varying sizes, those on proximal parts generally larger; merus triangular, about half length of ischium; palp (carpus, propodus and dactylus) shorter than merus, inserted on inner surface; dactylus distinctly longer than propodus; ischium subrectangular, with no visible median sulcus; basis-ischium and coxa not expanded, covered with numerous large rounded tubercles and some boletiform tubercles; exopod stout, broad, reaching to about half length of merus, basal part with large rounded, boletiform tubercles (Fig. 3D, E).

Chelipeds subequal, closely covered with tubercles of varying sizes and shapes; merus trigonal in cross-section, surface covered with closely-packed low, rounded granules, posterior margin with 3 or 4 large conical tubercles, inner margin with low, broad tubercles and granules; carpus rounded, surface covered with closely-packed low, rounded granules, inner distal angle with low tooth; palm short, stout, upper and outer surfaces with low, conical tubercles, relatively densely packed, inner surface prominently swollen, covered with large, rounded granules; fingers short, shorter than palm, dorsal margin of dactylus with row of low, short granules, outer surface with low, flattened granules, cutting edge with small sharp denticles, pollex relatively broader, outer surface with small rounded granules, submarginal ones arranged in approximate rows, cutting edge with low denticles (Figs 1, 2A, B, 3A, F).

Ambulatory legs short, decreasing in size posteriorly; merus, carpus and propodus covered with slender and boletiform tubercles along upper and lower margins, outer surface with large, rounded granules and tubercles; dactylo-propodal lock present; dactylus slender, glabrous, lined with granules, tip curved, corneous (Figs 2A, B, 3G).

Anterior thoracic sternites (1–4) strongly compressed; sternites 1 and 2 completely fused to form small plate; sternites 3 and 4 appearing fused but vaguely demarcated by arrangement of granules, surface of sternite 3 with numerous boletiform tubercles, larger on lateral surfaces; surfaces of sternites 4–7 with flattened and low, rounded tubercles (Figs 2B, 4A–D). Sternite 4 forming keel around distal part of sternopleonal cavity, occupying anterior third of overall cavity (Figs 3D, 4D). Sternopleonal cavity subovate, deeply excavated, glabrous, reaching buccal cavity anteriorly; sutures between sternites 4/5, 5/6, 6/7, and 7/8 interrupted medially (Figs 3D, 4D, E). Vulvae relatively small, round, positioned distinctly apart, with opening directed laterally (Fig. 4E).

Pleon ovate, shield-like, entirely covered with closely packed, rounded tubercles of varying sizes, larger ones partially coalescing; somite 1 very narrow, girdle-like, not visible when pleon closed, free; somite 2 narrow, semicircular, free; somites 3–6 completely fused, sutures not clearly visible in dorsal view, distinct in ventral view, margins lined with rounded granules; telson narrowly triangular, with proximal part broad, distal part linguiform, lateral margins deeply concave (Figs 2D, 4A–C).

##### Remarks.

Sakai (1980: 74) described the genus and species on the basis of only one 7.0 by 5.5 mm female, and no depth information was indicated with the provenance data. The description is relatively short and only one figure was provided. The whereabouts of the holotype is not known. We have checked the various museums in Japan, Germany, Netherlands and the USA where Sakai is known to have deposited material, but we could not locate the holotype in any of these countries. Sakai (1980: 73) commented that the source of his material was from the collection of Eiji Iishiba, a member of the Japanese Carcinological Society, and it is likely that the material was returned to Iishiba after study. Where the holotype is today is not known.

At 7.8 by 6.5 mm, the present ovigerous female from Taiwan is larger than the type but closely resembles it, except that the hepatic lobe is more plate-like (Figs 1B, 2A, C, D) (versus prominently granuliform); and the anterolateral lobes are more dentiform, the margins of each lobe less distinctly granuliform (Figs 1B, 2A, C, D) (versus lobes more rounded and distinctly lined with rounded granules) (cf. Fig. 1A; Sakai 1980: text-fig. 1).

Not considering the generic characters, the carapace of *Orientotlosiishibai* superficially most closely resembles that of *Aloxornatum* (Ihle, 1918), which also has many rounded granules on its surface. However, in *A.ornatum* the granules are arranged very differently, being more closely packed and sometimes coalescing (cf. Tan and Ng 1996: pl. 5A; Galil and Ng 2007: fig. 1C; Galil and Ng 2020: fig. 1A).

No males of *Orientotlosiishibai* have been collected, so the important characters of the male pleon and gonopods are unknown.

##### Biology.

Station CP4210 is a hard bottom habitat and the trawl net was seriously damaged, though its cod end was intact. That same haul contained many sponges and crinoids and their associated fauna, including a new species of stenopodid shrimp of the genus *Odontozona* Holthuis, 1946, often associated with sponges (Chen and Chan, in press). The hard substrate may explain the rarity of *Orientotlosiishibaii* in collections, as this habitat is very hard to sample, especially in deeper waters (see Mendoza et al. 2010). In fact, the rare deep-water western Pacific leucosiid *Galilia* Ng & Richer de Forges, 2007 originates from a similar habitat (see Ng and Richer de Forges 2007; Komai and Tsuchida 2014; Shih et al. 2015).

## Supplementary Material

XML Treatment for Orientotlos

XML Treatment for Orientotlos iishibai
